# Differential gene expression profiling reveals potential biomarkers and pharmacological compounds against SARS-CoV-2: Insights from machine learning and bioinformatics approaches

**DOI:** 10.3389/fimmu.2022.918692

**Published:** 2022-08-17

**Authors:** M. Nazmul Hoque, Md. Murshed Hasan Sarkar, Md. Arif Khan, Md. Arju Hossain, Md. Imran Hasan, Md. Habibur Rahman, Md. Ahashan Habib, Shahina Akter, Tanjina Akhtar Banu, Barna Goswami, Iffat Jahan, Tasnim Nafisa, Md. Maruf Ahmed Molla, Mahmoud E. Soliman, Yusha Araf, M. Salim Khan, Chunfu Zheng, Tofazzal Islam

**Affiliations:** ^1^ Department of Gynecology, Obstetrics and Reproductive Health, Bangabandhu Sheikh Mujibur Rahman Agricultural University, Gazipur, Bangladesh; ^2^ Bangladesh Council of Scientific & Industrial Research (BCSIR), Dhaka, Bangladesh; ^3^ Department of Biotechnology and Genetic Engineering, University of Development Alternative, Dhaka, Bangladesh; ^4^ Department of Biotechnology and Genetic Engineering, Mawlana Bhashani Science and Technology University, Tangail, Bangladesh; ^5^ Department of Computer Science and Engineering, Islamic University, Kushtia, Bangladesh; ^6^ National Institute of Laboratory Medicine and Referral Center, Dhaka, Bangladesh; ^7^ Molecular Bio-computation and Drug Design Laboratory, School of Health Sciences, University of KwaZulu-Natal, Durban, South Africa; ^8^ Department of Genetic Engineering and Biotechnology, School of Life Sciences, Shahjalal University of Science and Technology, Sylhet, Bangladesh; ^9^ Department of Immunology, School of Basic Medical Sciences, Fujian Medical University, Fuzhou, China; ^10^ Department of Microbiology, Immunology and Infectious Diseases, University of Calgary, Calgary, AB, Canada; ^11^ Institute of Biotechnology and Genetic Engineering (IBGE), Bangabandhu Sheikh Mujibur Rahman Agricultural University (BSMRAU), Gazipur, Bangladesh

**Keywords:** SARS-CoV-2, functional enrichment, gene regulatory networks, therapeutic targets, RNA-seq, genomics

## Abstract

The COVID-19 pandemic, caused by Severe Acute Respiratory Syndrome Coronavirus 2 (SARS-CoV-2), has created an urgent global situation. Therefore, it is necessary to identify the differentially expressed genes (DEGs) in COVID-19 patients to understand disease pathogenesis and the genetic factor(s) responsible for inter-individual variability and disease comorbidities. The pandemic continues to spread worldwide, despite intense efforts to develop multiple vaccines and therapeutic options against COVID-19. However, the precise role of SARS-CoV-2 in the pathophysiology of the nasopharyngeal tract (NT) is still unfathomable. This study utilized machine learning approaches to analyze 22 RNA-seq data from COVID-19 patients (n = 8), recovered individuals (n = 7), and healthy individuals (n = 7) to find disease-related differentially expressed genes (DEGs). We compared dysregulated DEGs to detect critical pathways and gene ontology (GO) connected to COVID-19 comorbidities. We found 1960 and 153 DEG signatures in COVID-19 patients and recovered individuals compared to healthy controls. In COVID-19 patients, the DEG–miRNA, and DEG–transcription factors (TFs) interactions network analysis revealed that E2F1, MAX, EGR1, YY1, and SRF were the highly expressed TFs, whereas hsa-miR-19b, hsa-miR-495, hsa-miR-340, hsa-miR-101, and hsa-miR-19a were the overexpressed miRNAs. Three chemical agents (Valproic Acid, Alfatoxin B1, and Cyclosporine) were abundant in COVID-19 patients and recovered individuals. Mental retardation, mental deficit, intellectual disability, muscle hypotonia, micrognathism, and cleft palate were the significant diseases associated with COVID-19 by sharing DEGs. Finally, the detected DEGs mediated by TFs and miRNA expression indicated that SARS-CoV-2 infection might contribute to various comorbidities. Our results provide the common DEGs between COVID-19 patients and recovered humans, which suggests some crucial insights into the complex interplay between COVID-19 progression and the recovery stage, and offer some suggestions on therapeutic target identification in COVID-19 caused by the SARS-CoV-2.

## Introduction

In late December 2019, a novel respiratory disease, now popularly termed “COVID-19”, caused by severe acute respiratory syndrome coronavirus 2 (SARS-CoV-2), emerged in Wuhan, China (1–3). Immediately after its first outbreak in China, this fearsome virus has emerged as one of the deadliest human pathogens (4, 5). As of June 22, 2022, COVID-19 disease affected 217 countries and territories, and more than 545 million cases have been confirmed around the globe, with more than 6.3 million deaths (6). Due to its worldwide spread and severity, the World Health Organization (WHO) has declared the disease a public health emergency of international concern (7–9). In the early stage of the outbreak, the spectrum of clinical manifestations of COVID-19 ranges from the common cold to respiratory failure depending on the demography and environment (2, 7, 10). However, recent data show that the clinical episodes of COVID-19 may range from asymptomatic infection to critical illness, with a dysregulated inflammatory response to infection a hallmark of severe cases (11) and life-threatening multiorgan failure (10, 12–14). In most cases (~80%), patients exhibit mild symptoms, while the remaining ∼20% may develop severe lung injury and death from respiratory failure (15–17). Some of the clinically infected patients may suffer from acute respiratory distress syndrome (ARDS) and multiple organ failures, requiring intensive care unit (ICU) facilities for life support and medication (16). Risk factors for severe SARS-CoV-2 include age, smoking status, ethnicity, and male sex (13, 18). Notably, the persistence and prognosis of COVID-19 are greatly influenced by the patients’ underlying health conditions and age (12, 19). With no effective antiviral treatment and slow vaccine rollout, COVID-19 continues to threaten public health worldwide seriously (20).

Despite increasing global threats of COVID-19, the host immune response against SARS-CoV-2 infection remains poorly understood, and the perturbations result in a severe outcome (15, 21). The nasal epithelium is a portal for initial infection and transmission of the SARS-CoV-2 (7). SARS-CoV-2 employs ACE2 (Angiotensin-converting enzyme 2) as a receptor for cellular entry (22, 23), and the binding affinity of the S protein and ACE2 was found to be a major determinant of SARS-CoV-2 replication rate and disease severity (21, 24). After the entrance into the susceptible host, SARS-CoV-2 infects cells of the respiratory epithelium and mucous membranes, such as those of the nose or eyes (22, 25). The host immune response to SARS-CoV-2 infection involves activation of both cellular and humoral arms. The innate immune system recognizes the SARS-CoV-2 RNAs through three major classes of cytoplasmic pattern recognition receptors: Toll-like receptors (TLRs), RIG-I-like receptors (RLRs), and NOD-like receptors (NLRs) (21, 26). This response involves the release of interferons (IFNs) and inflammatory cytokines, including the IL-1 family, IL-6, and TNF, that activates a local and systemic response to infection (7, 21). This inflammatory response cascade involves the recruitment, activation, and differentiation of innate and adaptive immune cells, including neutrophils, inflammatory myeloid cells, CD8 T cells, and natural killer (NK) cells (15). The infection resolution largely depends on the cytotoxic activity of CD8 T cells and NK cells, which enable the clearance of virus-infected cells (7, 21). It is believed that dysregulated host immune response leads to the persistence of virus-infected cells and may facilitate a hyper-inflammatory state termed Macrophage (MΦ) activation syndrome (MAS) or “cytokine storm”, and ultimately damage the infected lung (15, 21, 27). However, the underlying molecular mechanisms of the aberrant inflammatory responses in serology and histopathology under SARS-CoV-2 infection are still not clear.

The ongoing pandemic of SARS-CoV-2 and lack of comprehensive knowledge regarding the progression of COVID-19 has constrained our ability to develop effective treatments for infected patients. One way to understand the host response to SARS-CoV2 is to examine gene expression in relevant tissues. Until now, a scant amount of gene expression profiles are available from patients with COVID-19 and have yielded some insights into the pathogenic processes triggered by infection with SARS-CoV-2 (15, 21, 28). Transcriptomic analyses of cells upon viral infections are extremely useful for identifying the host immune response dynamics and gene regulatory networks (15, 29). However, because of the limited number of samples and preliminary analysis, a full picture of the physical state of SARS-CoV-2 affected tissues has not emerged. To address this, we have employed RNA-seq techniques to investigate the upper airway (nasopharyngeal tract) gene expression profile in 22 specimens of COVID-19 patients (n = 8), COVID-19 recovered (n = 7) and healthy (n = 7) individuals using several orthogonal bioinformatic tools to provide a complete view of the nature of the COVID-19 inflammatory response and the potential points of therapeutic intervention. Through DEG analyses in these datasets, we identified several genes coding for translational activities (e. g. RPL4, RPS4X, RPL19, RPS12, RPL19, EIF3E), ATP-synthesis (MT-CYB, MT-ATP6), transcription factors (e. g. E2F1, MAX, EGR1, YY1, SRF), hub-proteins (e. g. KIAA0355, DCUN1D3, FEM1C, ARHGEF12, THBS1), and mi-RNA (e. g. hsa-miR-19b, hsa-miR-495, hsa-miR-340, hsa-miR-101, and hsa-miR-19a) evidencing a sustained inflammation and cytokine storm in the COVID-19 patients.

## Materials and methods

### Ethical statement and consent of participants

his study was conducted following Bangladesh’s Director-General of Health Services (DGHS) guidelines. The protocol for a sample collection from COVID-19 recovered, and healthy humans, sample processing, transport, and RNA extraction was approved by the National Institute of Laboratory Medicine and Referral Center of Bangladesh. The study participants provided written informed consent consistent with the experiment.

### Study subject and sample collection

COVID-19 diagnosis, laboratory testing, and treatment in this cohort have been previously described (30). Patients with confirmed COVID-19 were classified as having mild/moderate (MM) or severe/critical (SC) disease based on symptomatology (22). We recruited seven recovered COVID-19 patients (post-hospital discharge) from this cohort and seven healthy subjects with no history of SARS-CoV-2 infection (negative control. Twenty-two (N = 22) nasopharyngeal samples (including COVID-19 = 8, recovered = 7, and healthy = 7) were collected from Dhaka city of Bangladesh. Collected samples were preserved at -20°C until further use for RNA extraction and RT-qPCR assay. The RT-qPCR was performed for *ORF1ab* and *N* genes of SARS-CoV-2 using a novel Coronavirus (2019-nCoV) Nucleic Acid Diagnostic Kit (PCR-Fluorescence Probing, Sansure Biotech Inc.) according to the manufacturer’s instructions. Viral RNA was extracted using a Pure Link viral RNA/DNA mini kit (Thermo Fisher Scientific, USA). Thermal cycling was performed at 50°C for 30 min for reverse transcription, followed by 95°C for 1 min, and then 45 cycles of 95°C for 15 s, 60°C for 30 s on an Analytik-Jena qTOWER instrument (Analytik Jena, Germany).

### RNA sequencing

We utilized the total RNA-seq approach for this study. According to the manufacturer’s instructions, the cDNA of all 22 samples was used to prepare paired-end libraries with the Nextera DNA Flex library preparation kit (Illumina, Inc., San Diego, CA). Paired-end (2 x 150 bp reads) sequencing of the prepared library pool of the samples was performed using a NextSeq high throughput kit with an Illumina NextSeq 550 sequencer at the Genomic Research Laboratory, Bangladesh Council of Scientific and Industrial Research (BCSIR), Dhaka, Bangladesh.

### Overview of the proposed bioinformatics pipelines

**Figure 1:**
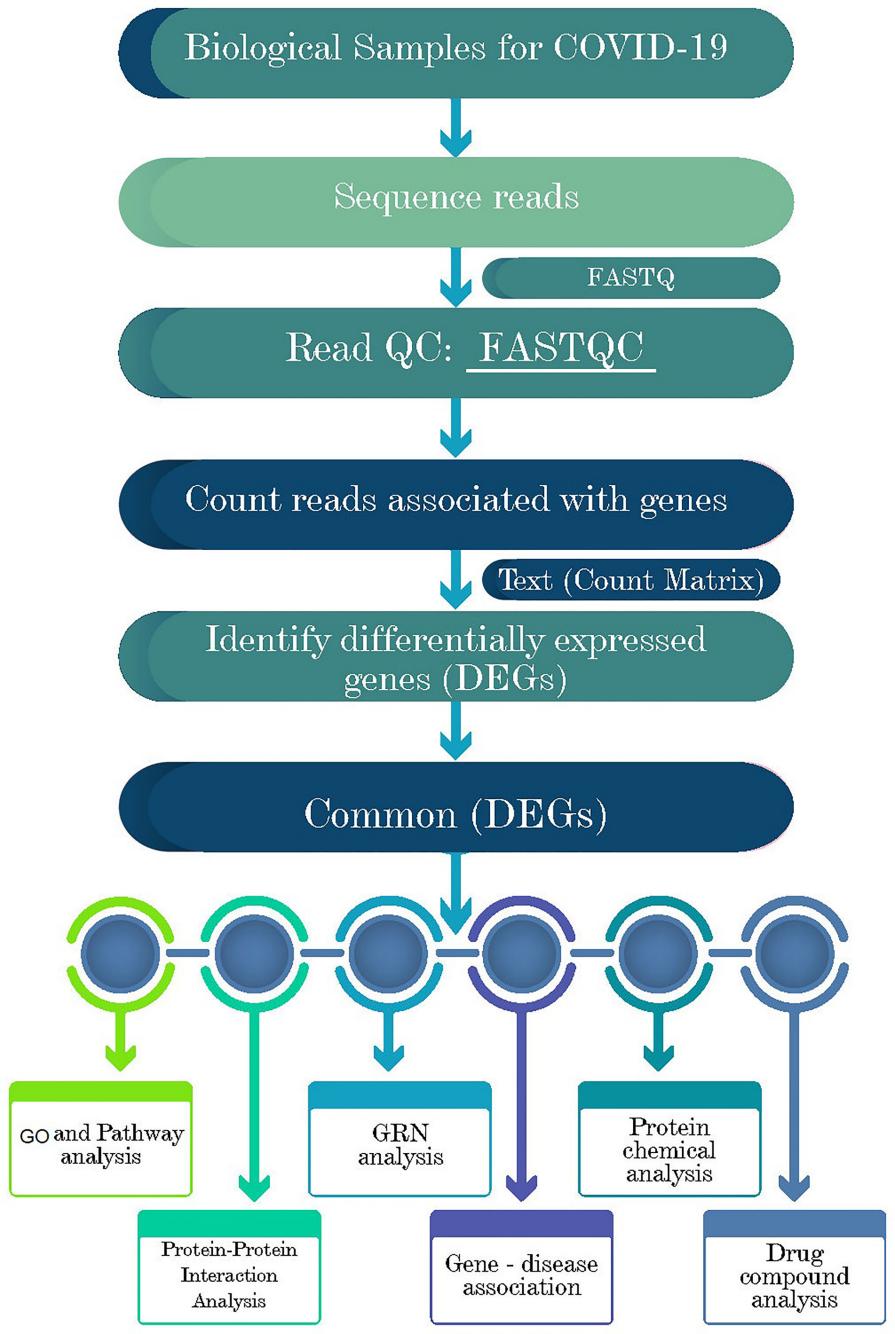
Schematic representations of the paths for differentially expressed genes (DEGs) analysis in RNA-seq data of the COVID-19 patients, recovered humans, and healthy controls nasopharyngeal tract.

Network-based approaches are common to identify and analyze the pathogenesis of SARS-CoV-2. Datasets required in this work were constructed and collected at the initial phase and detailed in the following subsections. Gene expression analysis was performed to identify the DEGs from each dataset (**Figure 1**). Next, the common DEGs between two groups of COVID-19 datasets were identified. These common DEGs were further used to discover their protein-protein interactions (PPIs) and to perform gene set enrichment analysis (GSEA) to identify enriched cell signaling pathways and functional gene ontology (GO) terms. Next, the same common DEGs were used to discover three types of GRNs: DEGs–micro RNAs (miRNA) network, DEGs–transcription factors (TFs) network, and TF-miRNA network. Finally, protein-chemical compound and protein-drug interactions were also investigated for the common DEGs (**Figure 1**).

### Dataset preparation and analysis of differentially expressed genes

To assess the DEGs of COVID-19 and their genetic association with host cells, we collected and analyzed the RNA-seq datasets from our lab experiment. In this study, we prepared two datasets as COVID-19 positive patients versus COVID-19 recovered humans with the same healthy control group for analytical purposes. We performed several statistical operations on the datasets to determine the DEGs. Moreover, the Benjamini–Hochberg false discovery rate method was used to balance the discovery of statistically significant genes and the limitation of false positives. The BioJupies generator (31) online server (https://maayanlab.cloud/biojupies/) was used for RNA-seq raw data analysis. In this study, genes with adjusted *P*-value < 0.05 and absolute value of log_2_ fold-change ≥ 1 were considered DEGs. Next, we compared two COVID-19 datasets to determine the shared DEGs using the Venny v2.1 web tool (32). In this article, we use the term combined DEGs’ to refer to the collection of these two sets of DEGs, which have been used in the downstream bioinformatics analyses.

### Functional enrichment analysis

We utilized Enrichr (33) with Fisher’s exact test to conduct the functional enrichment analysis with the combined DEGs. After performing an overrepresentation analysis, a collection of enriched cell signaling pathways and functional GO keywords were discovered, revealing the biological importance of the previously detected DEGs. In Enrichr analysis, we combined the signaling pathways from two libraries, including KEGG (Kyoto Encyclopedia of Genes and Genomes) and Reactome (https://reactome.org/), to create a single route. Only the important paths for which the *P*-value was less than 0.05 were evaluated and considered after deleting duplicate pathways. For functional GO annotations, we looked at the GO biological process, GO molecular function, and GO cellular component datasets in Enrichr and selected the most important GO terms based on set criteria and with an adjusted *P*-value < 0.05.

### Protein-protein interaction network analysis

Protein-protein interaction (PPI) of the shared DEGs was analyzed using the STRING database (34). We applied different local- and global-based methods using the cytoHubba plugin in Cytoscape v3.8.2 (35) to determine potential hubs within the PPI network. While the local method ranked hubs based on the relationship between the node and its direct neighbor, the global method ranked hubs based on the interaction between the node and the whole network. In total, five different methods were considered, including three local rank methods, i.e., degree, maximum neighborhood component (MNC), maximal clique centrality (MCC), and two global rank methods, i.e., edge percolated component (EPC) and betweenness. Next, we compared the results and identified the common nodes as the most potential hubs. Finally, the protein networks were analyzed through Cytoscape v3.8.2.

### Differential gene regulatory network analysis

The findings of DEG–miRNA, TF–DEG, and TF-miRNA interaction networks are part of the GRN analysis. Using the Network Analyst platform (36), the commonly dysregulated genes were utilized to identify GRN networks. Discovering DEG–miRNA interaction networks was accomplished through the miRTarBase database (37). To identify the TF–DEG interaction network, the JASPAR database (38) was used. Employing TF-miRNA coregulatory network database, the TF-miRNA interaction was analyzed. The networks were filtered with a betweenness value of 100 and degree centrality of 0 to 10 to remove unnecessary information.

### Protein–chemical compound analysis

Analyses of protein–chemical compounds can be used to identify the chemical molecules responsible for the interaction of proteins in comorbidities. For example, this study found protein–chemical interactions using the enriched gene (common DEGs) that COVID-19 patients developed several digestive problems. Furthermore, using the Comparative Toxicogenomics Database (39), we have identified the protein–chemical interactions through Network Analyst (36).

### Protein–drug interaction network

One of the key goals of this study is to identify potential therapeutic compounds that could effectively mitigate SARS-CoV-2 pervasiveness. Using the shared DEGs, we constructed the protein-drug interaction (PDI) network through the Network Analyst v3.0 web server (36) in conjunction with the DrugBank v5.0 database (https://go.drugbank.com/docs/drugbank_v5.0.xsd). To aid the analysis, we downloaded the network data and configured the data with Cytoscape v3.8.2 (35).

### Gene-disease association prediction

DisGeNET (https://www.uniprot.org/database/DB-0218) is a standardized gene-disease association database that incorporates correlations from various sources involving various biological features of disorders (40). It emphasizes the increasing understanding of human genetic illnesses. We examined the gene-disease connection using a network analyzer (36) to find diseases and chronic problems associated with common DEGs.

## Results

### Differentially expression and distribution of DEGs

**Figure 2:**
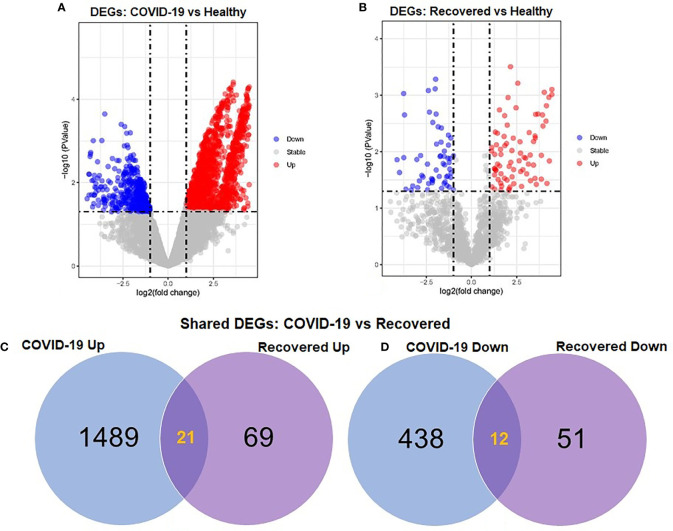
Volcano plots showing dysregulated genes in **(A)** COVID-19 patients vs. healthy control and **(B)** recovered humans vs. healthy controls. The red and blue dots indicate the expressions of the upregulated (Up) and down-regulated (Down) DEGs, respectively. Venn diagrams depict the unique and shared DEGs **(C)** upregulated and **(D)** down-regulated under the given conditions.

**Figure 3:**
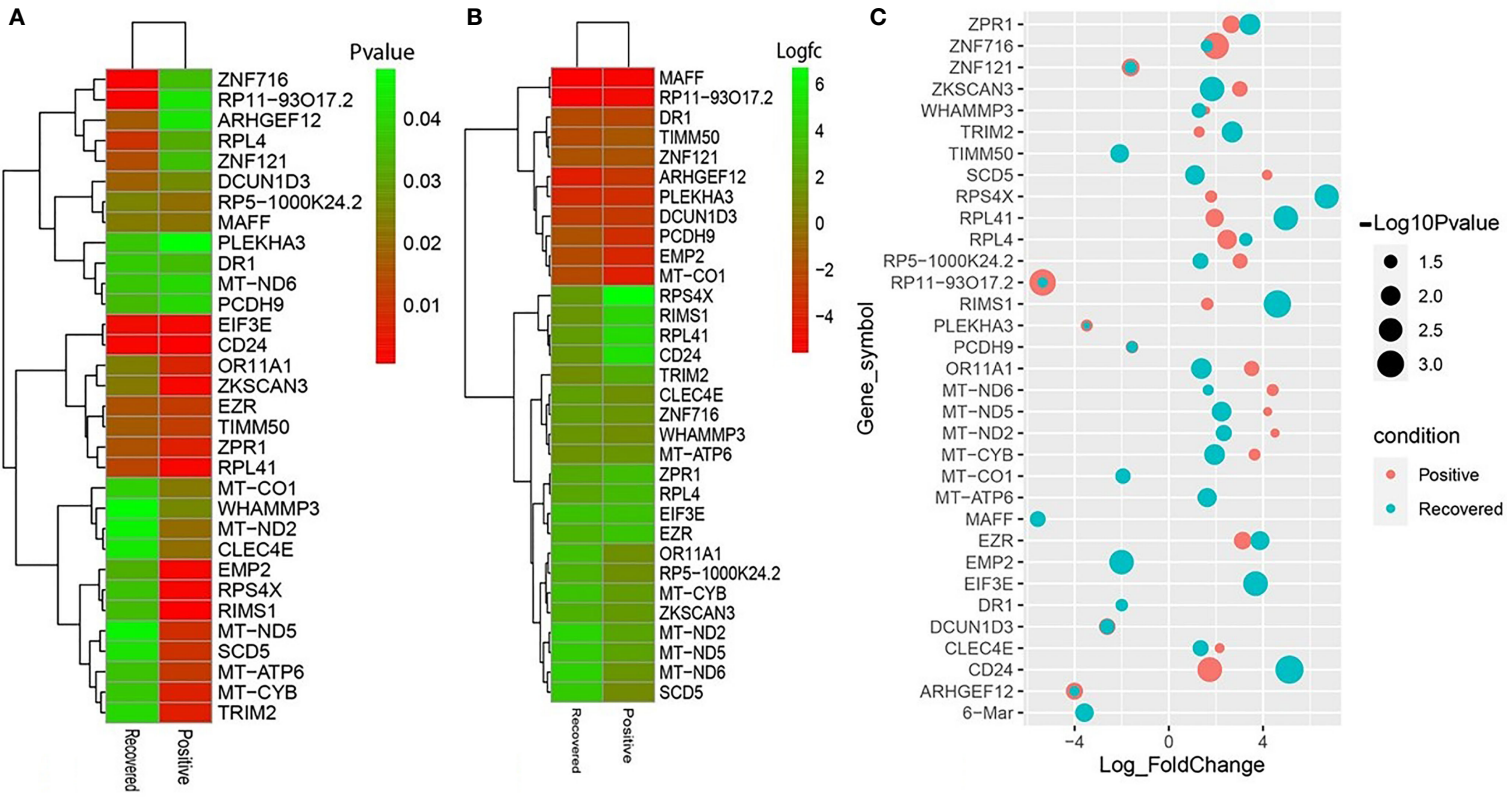
Heatmaps depicting the relationships among common DEGs in COVID-19 patients and recovered subjects based on **(A)** adjusted *p*-value and **(B)** logFC values. **(C)** Bubble plots showing the combined Log_10_ fold-changes and *p*-values for the shared common genes between COVID-19 patients and recovered humans. The red color indicates the genes of COVID-19 patients, and the green color presents the genes of the recovered people, while the mixed color indicates the overlapping genes.

To elucidate whether differentially expressed genes (DEGs) contribute to the SAR-CoV-2 inflammatory response and the potential points of therapeutic intervention, we analyzed 22 RNA-seq data of nasopharyngeal epithelial tissue of COVID-19 patients, recovered humans, and healthy controls. To perform RNA-seq analysis, we retrieved datasets from the National Center for Biotechnology Information (NCBI) that belonged to previously published BioProject under accession number PRJNA720904 (https://www.ncbi.nlm.nih.gov/bioproject). We identified 1960 and 153 gene signatures in COVID-19 patients and recovered human NT epithelial tissues, which were differentially expressed compared with healthy controls. We particularly focused on the dysregulation (up or down-regulation) of the identified DEGs during SARS-CoV-2 pathophysiology and its overlap with the recovered or healthy states of the humans. The volcano plots in **Figure 2** show the DEGs for COVID-19 with the red dots. The number of shared DEGs between COVID-19 and recovered datasets is presented in the Venn diagrams (**Figures 2C**, **D**). Thirty-seven shared DEGs were identified between COVID-19 patients and recovered subjects. Of the detected DEGs, 1,510 (77.04%) genes were upregulated (Up) during SARS-CoV-2 pathogenesis, of which 1,489 (98.61%) genes had a sole association with COVID-19 patients. Likewise, 90 (58.82%) genes were upregulated in recovered humans, and of them, 69 (76.67%) genes had a sole association with the recovery phage of SARS-CoV-2 infection (**Figure 2C**). By comparing the upregulated genes between COVID-19 patients and recovered individuals, we found that 21 genes (i.e., RPL4, MT-ND2, SCD5, MT-CYB, EZR) were shared between the conditions (**Figure 2C**). On the other hand, 450 (22.96%) and 63 (41.18%) DEGs were downregulated (Down) in COVID-19 patients and recovered subjects, respectively, and of them, only 12 genes (i.e., MAFF, ARHGEF12, DCUN1D3, DR1, MT-CO1.) were found to be shared between COVID-19 and recovered cases (**Figure 2D**). The DEGs shared between COVID-19 positive and recovered people and their relationships from the perspective of adjusted *P*-value, and log2 fold-change is presented in the heatmaps, respectively (**Figures 3A**, **B**). Finally, 33 common dysregulated (Up or Down) genes were presented in a bubble plot to show relationships with 10 log fold-change values (**Figure 3C**).

### Functional enrichment analysis identifies significant cell signaling pathways and gene ontology

**Figure 4:**
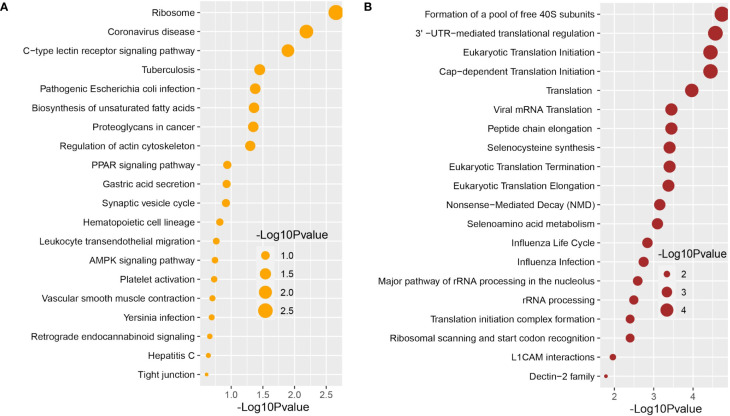
Signaling pathway analysis of nasopharyngeal epithelial cells on COVID-19 patients. We find that the top 20 terms depend on the *P*-value. **(A)** KEGG pathway and **(B)** Reactome pathway.

We used the Enrichr tool to conduct a functional enrichment analysis on the DEGs to identify the signaling pathways and functional GO keywords significantly enriched with DEGs in the nasopharyngeal epithelial cells from COVID-19 patients. The 33 shared DEGs were used to identify key pathways and GOs that may be linked to COVID-19 comorbidities. We combined the KEGG and Reactome pathway databases with Enrichr tools to create a single pathway database. We looked at the pathways whose significance was determined by the *P*-value and plotted the top 20 pathways for each condition (**Figure 4**). Consideration was given to the paths having a higher logarithmic *P*-value. The most significant pathways were the ribosome signaling pathway, coronavirus signaling pathway, and c-type-lectin receptor signaling pathway for KEGG analysis (**Figure 4B**) and forming a pool of free 40S subunits 3-UTR-mediated translational regulation, and eukaryotic translational initiation signaling pathways for the Reactome database (**Figure 4B**).

We used the Enrichr tool to identify significantly enriched cellular signaling pathways and functional GO terms (molecular function, biological process, and cellular component) with DEGs in the nasopharyngeal epithelial cells from COVID-19 patients. The 33 shared DEGs were used to identify key pathways and GOs that may be linked to COVID-19 comorbidities. We looked at the pathways whose significance was determined by the *P*-value (having a higher logarithmic *P*-value) and plotted the top 20 pathways for each condition (**Figure 4**).

**Figure 5:**
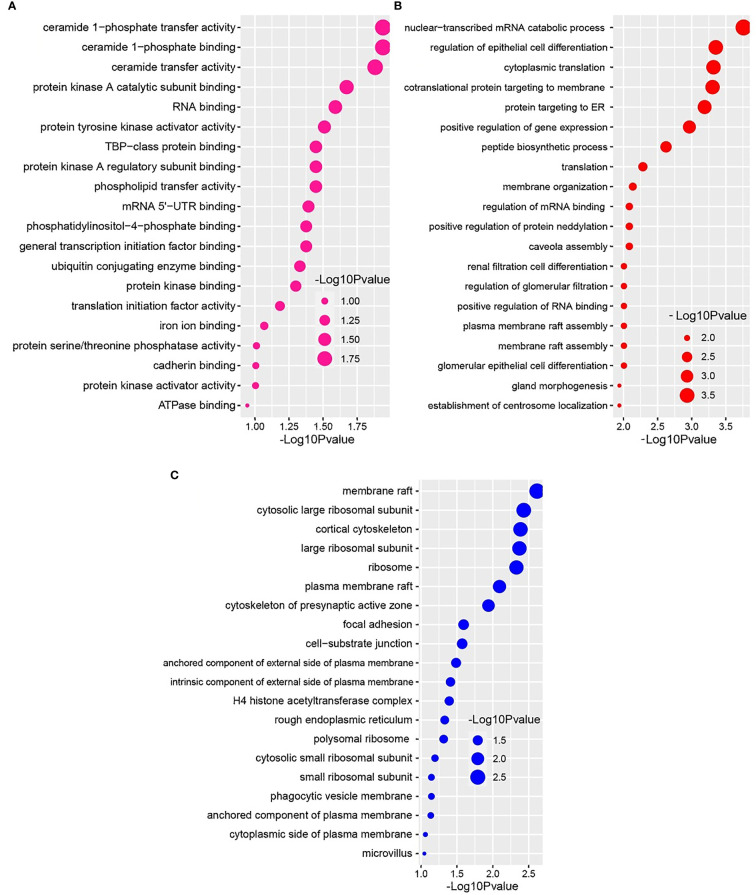
Based on the adjusted *P*-value, the top 20 cell signaling pathways in the nasopharyngeal epithelial cells in COVID-19 patients. The pathways have been formed by combining the DEGs that are common in the **(A)** gene ontology (GO) molecular function, **(B)** GO biological process, and **(C)** GO cellular component.

We further conducted GO functional enrichment analysis using the same common DEGs. We employed the GO biological process, the GO molecular function, and the GO cellular component databases obtained from Enrichr libraries. The significantly enriched GO terms were identified if the enrichment yielded the adjusted *P*-value’s high logarithmic value. The top 20 cellular signaling pathways in the COVID-19 patient’s nasopharyngeal epithelial cells were selected in this study (**Figure 5**) in relevance to the recovered phase. The most significant GO pathways were the ceramide 1-phosphate transfer activity, and ceramide 1-phosphate binding pathways for the molecular functions (**Figure 5A**), database nuclear-transcribed mRNA catabolic process and regulation of epithelial cell differentiation pathways for biological process (**Figure 5B**), and membrane raft, and cytosolic sizeable ribosomal subunit pathways for cellular component (**Figure 5C**).

### Protein-protein interaction network construction and interaction analysis

**Figure 6:**
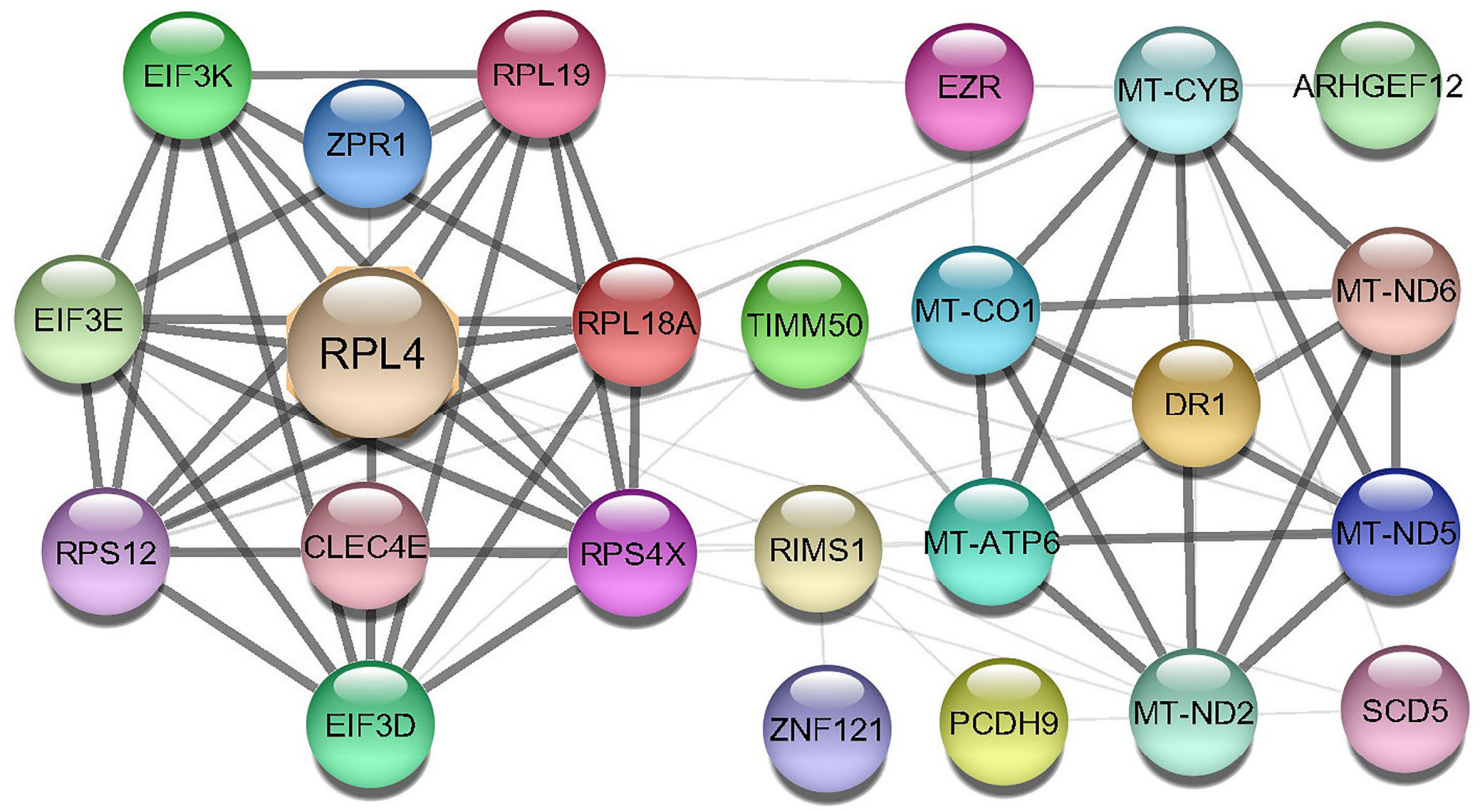
Protein-protein interaction (PPIs) network of common DEGs in COVID-19 patients. The nodes represent the proteins, and the edges represent the interactions across the proteins. Proteins having more edges are highly expressed, and thickness between the edges indicates the strength of interactions.

**Figure 7:**
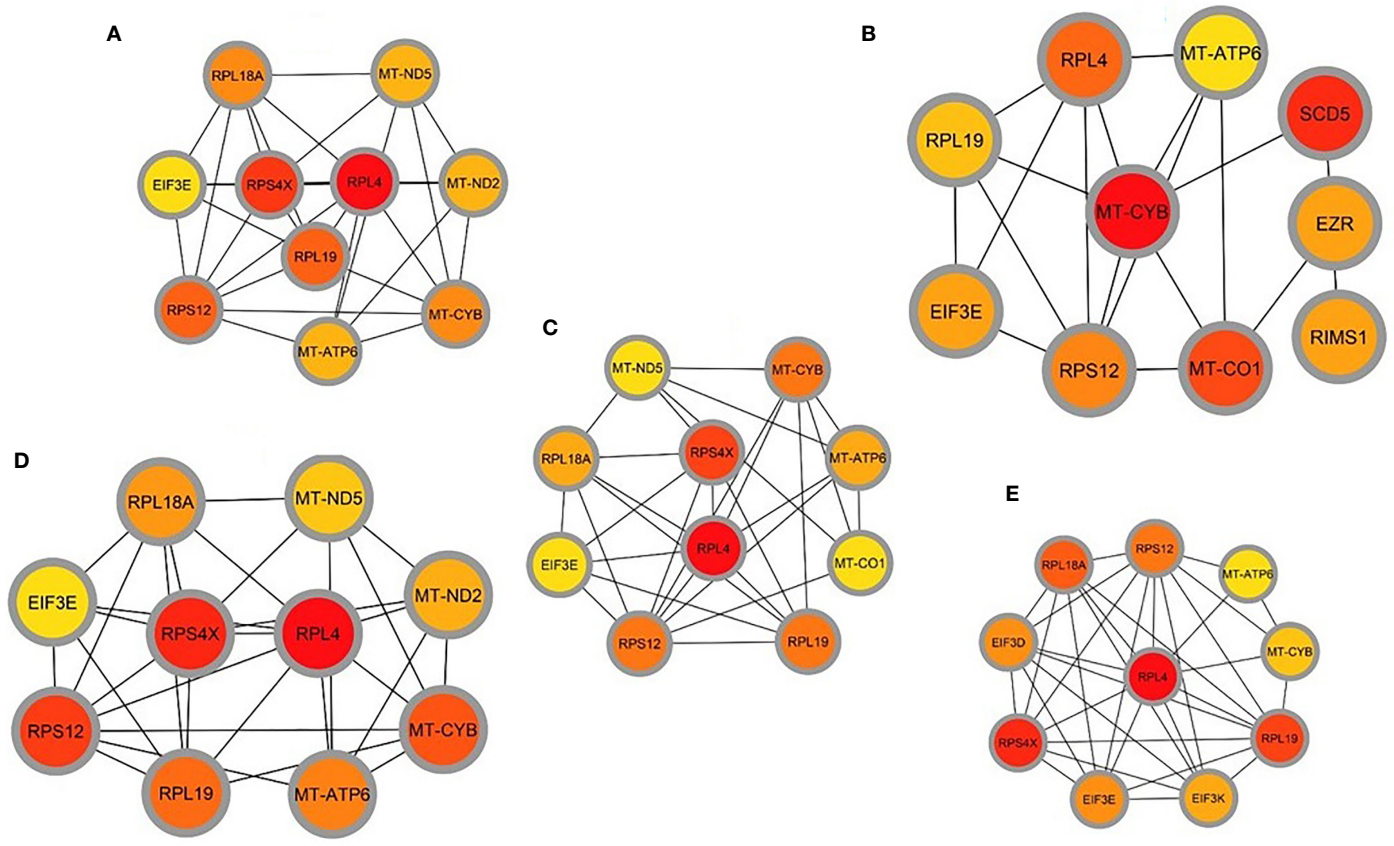
Determination of hub genes from the protein-protein interaction (PPI) network by using the Cytohubba plugin in Cytoscape. We applied five algorithms of the Cytohubba plugin to obtain the hub genes. Here **(A)** maximum neighborhood component (MNC), **(B)** betweenness, **(C)** degree, **(D)** edge percolated component (EPC), and **(E)** maximal clique centrality (MCC). Red to yellow color gradients indicate the higher ranking of hub genes.

A Protein-protein interaction (PPI) network was built from the common DEGs interactions, consisting of 24 nodes and 72 edges. The PPI network clustering highlighted RPL4, RPL18A, EIF3E, EIF3D, RPS4X, RPL19, EIF3K, RPS12, MT-ND2, MT-CO1, MT-ATP6, and MT-CYB with high interaction activity (**Figure 6**). The proteins with several connecting edges can be identified as hub proteins. **Figure 7** shows the top 10 hub nodes within the PPI network. As anticipated by five different methods (i.e., maximum neighborhood component; MNC, betweenness, degree, edge percolated component; EPC and maximal clique centrality; MCC), we recognized eight hub-nodes as potential hub-proteins (i.e., RPL4, RPS4X, RPL19, RPS12, RPL19, EIF3E, MT-CYB, and MT-ATP6) (**Figures 7A**–**E**). Interestingly, these eight hub proteins were common in all methods. Only RPS4X was found from 4 methods except for betweenness (**Figure 7B**). Conversely, the betweenness method predicted only three proteins (i.e., SCD5, EZR, and RIMS1) from the shared DEGs as hubs that were not found by other methods (**Figure 7B**).

### GRN analysis identifies DEGs–miRNA and transcription factor–gene interactions

**Figure 8:**
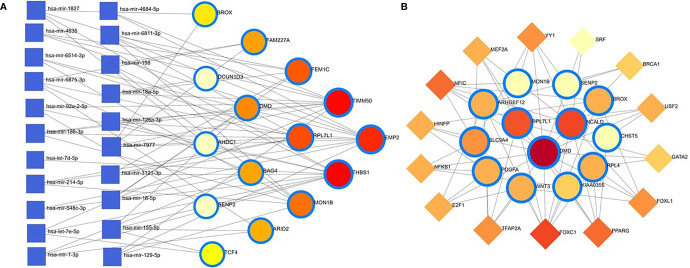
Genes’ regulatory networks. **(A)** Gene regulatory networks (gene-miRNA) of the nasopharyngeal epithelial cell in COVID- 19 patients with the shared dysregulated genes. The square shapes and circular shapes represent the miRNA and genes, respectively. **(B)** Gene regulatory networks (transcription factors; TF) of the nasopharyngeal epithelial cell in COVID- 19 patients with the shared dysregulated genes. The square shapes and circular shapes represent the TF and genes, respectively.

The common DEGs between COVID-19 patients and recovered humans were used in this study. The DEG–miRNA interactions network is depicted in **Figure 8A**. The dysregulated genes are shown by the circles in the picture, while the squares represent the miRNAs. The association among different nodes of DEGs and miRNA (circles or squares) is represented by different lines linking them. Significant nodes are those in a network that connect several edges because they are more crucial. Out of 21 miRNAs detected, hsa-let-7e-5p, hsa-mir-7977, hsa-mir-155-5p, hsa-mir-186-5p, and hsa-mir-1827 were the most expressed miRNAs and had a stronger association with DEGs (**Figure 8A**). Likewise, among the DEGs, DMD, AHDC1, BAG4, EMP2, TIMM50, RPL7L1, and THBS1 were more significant since these DEGs have a higher degree (number of connecting edges) than the others and miRNAs (**Figure 8A**). We further studied the interactions between TF and DEGs and identified 14 TFs, of which FOXC1, FOXL1, NFIC, YY1, and PPARG were significantly enriched and showed more interactions with DEGs (**Figure 8B**).

**Figure 9:**
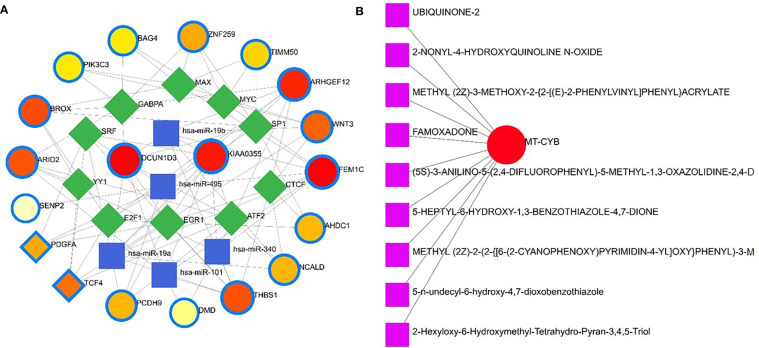
Gene regulatory and protein-drug interactions. **(A)** Gene regulatory networks (TF-miRNA) of the nasopharyngeal epithelial cell in COVID- 19 patients with the shared dysregulated genes. The blue color and square shapes indicate the miRNA, while the green color is square-shapes, representing TF. **(B)** Protein-drug interaction network. Nine pharmacological compounds are indicated by squares (pink color), while one circle shape (red) represents the hub node.

Apart from these, the present study included TFs and miRNAs highly relevant to SARS-CoV-2 interactions. This analysis identified 19 hub proteins, 10 TFs, and 5 miRNAs (**Figure 9A**). In COVID-19 interaction, the TF–miRNA network showed that E2F1, MAX, EGR1, YY1, and SRF were the highly expressed TFs, and hsa-miR-19b, hsa-miR-495, hsa-miR-340, hsa-miR-101, and hsa-miR-19a were among significant miRNAs (**Figure 9B**).

### Protein-drug and protein-chemical interactions reveal possible drugs for COVID-19 patients

Protein-drug interaction (PDI) networks provide a wealth of information about possible pathogenesis mechanisms and drug interactions that may not be evident using conventional approaches. To disrupt the SARS-CoV-2 pervasiveness, we sought to find pharmaceutical compounds that interact with viral proteins (Methods). We detected nine pharmacological compounds (for example, famoxadone, ubiquinone-2, 2-nonyl-4-hydroxyquinoline, 5-n-undecyl-6-hydroxy-4,7-dioxobenzothiazole) acting against one protein, the human mitochondrial cytochrome b (MT-CYB) (**Figure 9B**).

**Figure 10:**
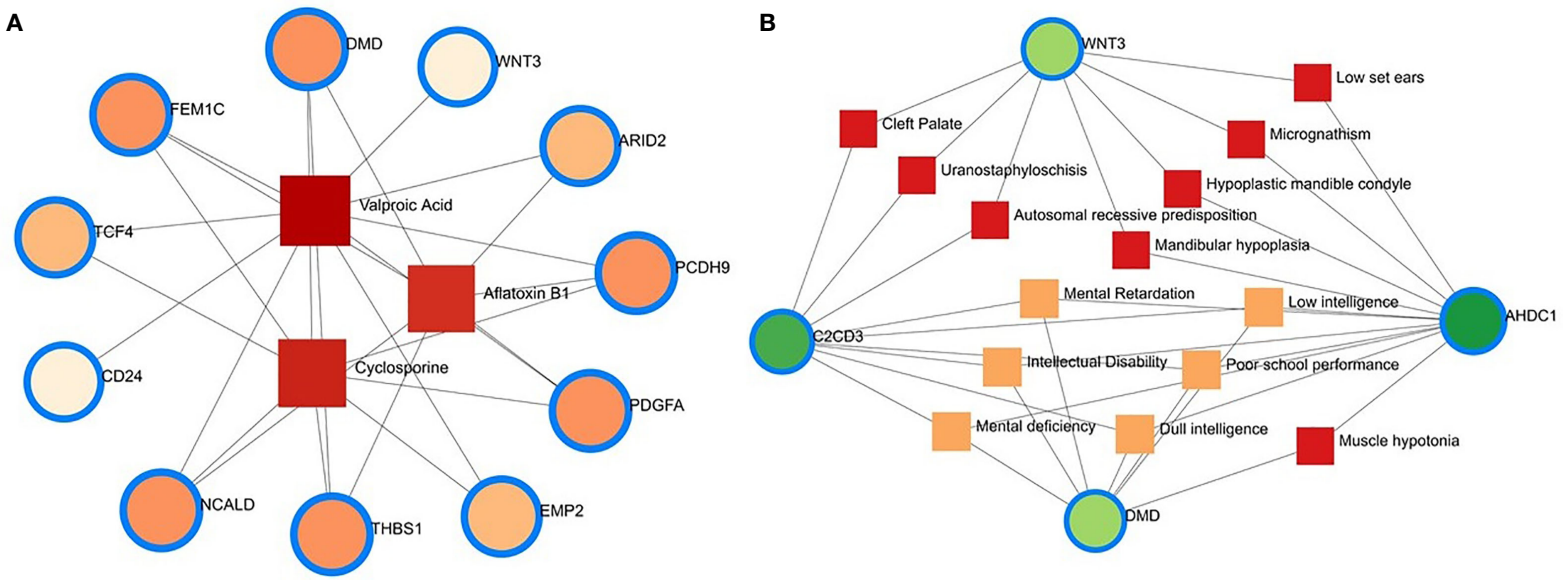
Protein-chemical and gene-disease association. **(A)** Protein-chemical interaction network. Three phytochemical compounds were found against 11 genes. Circles showed the shared DEGs, while square shapes indicated interacting phytochemical compounds. **(B)** Gene-disease association network. Circles indicate the common differential genes, and the 14 square shapes represent the common diseases interconnected to the COVID-19 patients.

Protein–chemical interaction (PCI) is an important study to understand the functionality of proteins underpinning the molecular mechanisms within the cell, which may also help in drug discovery. For example, it has been discovered that SARS-CoV-2 infection causes PCI networks in the COVID-19 patients and recovered humans. **Figure 10A** depicts a network of PCI among significant proteins. The significant proteins identified from this network include FEM1C, NCALD, THBS1, PCDH9, DMD, and PDGFA. Similarly, we identified three chemical agents, Valproic Acid, Alfatoxin B1, and Cyclosporine, enriched in this interaction analysis (**Figure 10A**).

### Gene-disease network finds different diseases associated with COVID-19

This study hypothesizes that many conditions can be associated or connected with COVID-19 by sharing some common genes. Disorder-specific therapeutic interface strategies attempt to discover the link between genes and diseases. In this study, we found 14 other diseases associated with COVID-19 by sharing four DEGs (i.e., DMD, C2CD3, WNT3, and AHDC1) most prevalent in COVID-19. Of the detected diseases, mental retardation, mental deficiency, intellectual disability, muscle hypotonia, micrognathism, and cleft palate were the significant diseases interconnected with COVID-19 (**Figure 10B**).

## Discussion

The SARS-CoV-2 infection causes a wide spectrum of diseases ranging from minimal, often asymptomatic, respiratory illness to severe pneumonia with multisystem failure and death. The ongoing rapid transmission and global spread of COVID-19 have raised intriguing questions whether the evolution and adaptation of SARS-CoV-2 is driven by changes at the gene levels (41). Therefore, this work investigates the influences of SARS-CoV-2 infection on differential gene expressions (DEGs) in the nasopharyngeal epithelial cells of the COVID-19 patients and recovered individual. We identified 1960 and 153 DEGs in COVID-19 patients and recovered humans with different expressions than healthy controls. Among these DEGs, 77.0% were upregulated during SARS-CoV-2 pathogenesis, and more than 98.0% of the upregulated gene signature had a sole association with COVID-19 patients. Therefore, relatively higher genes were upregulated in COVID-19 patients compared to recovered and healthy humans. Earlier studies reported that certain differences in gene expression between patient groups might be driven by changes in tissues’ cellular composition, including through the recruitment of immune cell types to the site of infection (14). By analyzing the RNA-seq dataset of lung epithelial cells infected with SARS-CoV-2, Jha et al. (42) identified 338 DEGs, including 92 increased and 246 decreased genes across the datasets. In this study, top abundant DEGs such as genes encoding for ribosomal protein (RPL4), controlling the production of the mitochondrial reactive oxygen species (MT-ND2) (43), modulating cell proliferation and differentiation (SCD5) (44), mitochondrial deficiencies and associated disorders (MT-CYB) (45), epithelial marker ezrin (EZR) associated with cell surface structure adhesion, migration and organization of the SARS-CoV-2 (46) were found to be co-expressed in the nasopharyngeal epithelial cells of COVID-19 patients and recovered humans (**Figure 2C**). Conversely, SARS-CoV-2 infection suppressed the expression of genes associated with transcription factors (MAFF) (47), erythropoiesis (ARHGEF12) (48), membrane neddylation (DCUN1D3) (49), a global regulator of transcription (DR1) (50), and cytochrome-c oxidase activity (MT-CO1) (51) in both COVID-19 patients and recovered humans (**Figure 2D**).

We next investigated whether host gene expression during SARS-CoV-2 pathophysiology is associated with functional enrichment, for example, cell signaling pathways and gene ontology. Our results showed that DEGs related to ribosome signaling pathway, coronavirus signaling pathway, c-type-lectin receptor signaling pathway, forming a pool of free 40S subunits, 3-UTR-mediated translational regulation, and eukaryotic translational initiation signaling pathways were significantly enriched in the nasopharyngeal epithelial cells on COVID-19 patients. These findings corroborated with the previously published studies conducted to understand host transcriptional response to influenza A virus and SARS-CoV-2 in primary human bronchial epithelial cells (28, 42). Gene ontology analysis identified several pathways: ceramide 1-phosphate transfer activity, ceramide 1-phosphate binding pathways, nuclear-transcribed mRNA catabolic process, regulation of epithelial cell differentiation pathways for biological process, membrane raft and cytosolic sizeable ribosomal subunit pathways for cellular component significantly enriched in COVID-19 patients. Ceramide 1 phosphate (C1P) can augment immunity and control COVID-19 infection by enhancing autophagy, adaptive immunity (Th1 programming), and MHC-I-dependent cytotoxic T lymphocytes (CTL) response (52). The epithelium lining the airways plays a key role in the defense against infections. Several lines of evidence showed that SARS-CoV-2 infection induces epithelial barrier function, as documented by decreased trans-epithelial resistance, increased permeability, and altered tight junction protein distribution (53, 54). However, this functional impairment remained transient, with signs of epithelial regeneration during the recovery phage of SARS-CoV-2 infection. Basal cell mobilization and replication can also be observed to exert a moderate effect on epithelial barrier integrity (53). With these dysregulated genes, we have conducted the PPI network analyses. PPIs network analysis is the most prominent section of the study as hub gene detection, analysis of modules and drug identification thoroughly depends on the PPIs network. According to the PPIs network (**Figures 6**, **7**), ribosomal proteins (RPL4, RPS4X, RPL19, RPS12, RPL19), translation initiation factor 3 subunit E (EIF3E), mitochondrial deficiencies and associated disorders (MT-CYB) (45), and cytochrome-c oxidase activity (MT-CO1) (51), and mitochondrial oxidative phosphorylation (MT-ATP6) proteins were declared as hub genes because of their high interaction rate or degree value. SARS-CoV-2 infection regulates the mitochondrial transcription of the proteins (MT-CO1 and MT-ATP6) involved in ATP synthesis, respiratory activity, oxidative stress, pro-inflammatory state, and cytokine production (55). The increased expression of ribosomal proteins can be attributed to the virus hijacking the host’s translational machinery for its survival by the mechanisms such as ribosome shunting and phosphorylation of ribosomal proteins (42). The PPI and gene enrichment analyses of these hyper-interactive proteins showed significant biological functions connected to COVID-19 related to the cell signaling pathway and the host response to SARS-CoV-2 infections (56). As discussed earlier, these proteins are involved in several other disorders (28, 55, 56).

We further studied relationships of the common DEGs between COVID-19 patients and recovered humans concerning protein-protein, gene-miRNA, TF-gene, protein-drug, and protein-chemical interactions. Our results showed that hsa-miR-19b, hsa-miR-495, hsa-miR-340, hsa-miR-101 and hsa-miR-19a were the mostly expressed miRNAs (**Figure 8A**), and E2F1, MAX, EGR1, YY1 and SRF were the highly expressed transcription factors (TFs) (**Figure 8B**). While host responses to infection are critical in differential outcomes of SARS-CoV-2 infection, the role of miRNAs in COVID-19 pathogenesis is poorly understood. We observed that most of these miRNAs were strongly upregulated in COVID-19 patients, which could be used as the circulating biomarkers for the diagnosis or prognosis of COVID-19 (57). Circulating miRNAs are extracellular serum/plasma miRNAs that could be involved in cell-cell communication and might contribute to disease progression. Besides their diagnostic value, miRNAs are well known for their therapeutic potential, especially in viral diseases. A recent report has compared the miRNA signature in the peripheral blood of COVID-19 patients versus healthy donors and several miRNAs have been identified to be deregulated, and interfered with the shaping of the immune responses (58). Therefore, the upregulated levels of miRNAs could be involved in the inflammatory storm seen in COVID-19 patients by inhibiting the immunosuppressive and anti-inflammatory role ensured by the transcription signaling pathway. Recent studies reported that transcription of mRNAs in epithelial cells is induced by TNF-α and triggers a negative feedback loop involving E-selectin to control inflammatory signaling (42, 59). Although we identified the 14 TF-genes showing more interactions with DEGs, we have to assess further whether these genes have the potential causal effects on the COVID-19 development. Our network-based approach identified TF hubs that likely regulate many cellular functions (e.g., cytokine storm) overexpressed in COVID-19 patients. Previous research identified 95 TFs in cytokines upregulated in the COVID-19 patients, and of them, 19 TFs are targets of FDA-approved drugs (60). Targeting TFs associated with the cytokine-releasing syndrome provides candidate drugs and targets to treat COVID-19 (60). However, additional research is needed to determine whether these combinations elicit the same immunomodulatory response in the context of SARS-CoV-2 infection.

Nine pharmacological compounds were found to be effective against SARS-CoV-2, and of them, fungicide (famoxadone), blood pressure controlling coenzyme (ubiquinone-2), secondary metabolite producing quinoline (2-nonyl-4-hydroxyquinoline), and xenobiotic compound (5-n-undecyl-6-hydroxy-4,7-dioxobenzothiazole) showed their activity against the human mitochondrial cytochrome b (MT-CYB). These compounds have significant antimicrobial, antidiabetic, anti-inflammatory, antiviral, and antioxidant activities (61) against SARS-CoV-2 infections. Furthermore, protein-chemical interaction (PCI) showed that FEM1C, NCALD, THBS1, PCDH9, DMD, and PDGFA proteins interacted with three chemical agents such as Valproic acid (VPA), Alfatoxin B1, and cyclosporine. Numerous promising antiviral therapies against SARS-CoV-2 are being investigated to prevent interindividual transmission and severe complications of the COVID-19. The VPA can reduce the SARS-CoV-2 receptor ACE-2 expression level and can be used as a potential drug candidate for the prevention strategy against COVID-19 (62). Aflatoxin B1 (AFB1), which alters immune responses to mammals, is one of the most common mycotoxins in feeds and food and a potential aggravating risk factor in COVID-19 patients (63). The effect of cyclosporine on coronaviruses, including the new SARS-CoV2, has been extensively studied (64). Several earlier studies showed that cyclosporine has the potential to prevent uncontrolled inflammatory response, SARS-CoV-2 replication, and acute lung injury (64, 65). Therefore, effective drugs are urgently needed to target this life-threatening complication, particularly for patients developing acute respiratory distress syndrome. In addition, we identified 14 other diseases associated with COVID-19 by sharing four DEGs (i.e., DMD, C2CD3, WNT3 and AHDC1) which were most prevalent in COVID-19. People with SARS-CoV-2 infections often have coexisting conditions like mental retardation, mental deficiency, intellectual disability, muscle hypotonia, micrognathism, and cleft palate. There is a dearth of information regarding the impact of COVID-19 in patients with tuberculosis, HIV, chronic hepatitis, and other concurrent infections (66). COVID-19 patients developed serious symptoms, including difficulty breathing, chest pain, loss of muscle control, severe inflammation, and organ damage. The adverse health and economic impact of the COVID-19 pandemic influenced mental health, causing distress, anxiety, and depression (67). These complications are not necessarily short-lived and can cause long-term effects of multiorgan injury following SARS-CoV-2 infections. COVID-19 presents a greater risk to people with intellectual and developmental disabilities, especially younger ones, and recent evidence suggests that mental health problems significantly increased worldwide during this pandemic (68, 69). Since muscle possesses the ACE2 receptor to which SARS-Cov-2 binds, it follows that the involvement of the muscle could be due not only to the secondary effects of the infection (e.g., reduced oxygen supply from persistent lung disease, perfusion defects from cardiovascular defects and vascular damage), but also to the direct action of virus (SARS-CoV-2 myositis) (70).

## Conclusions

Gene expression analysis may potentially reveal disease-pathogenesis pathways and point to novel targets for potential therapeutic approaches. This study examines the RNA-seq data of COVID-19 patients, recovered persons, and healthy individuals to find DEGs and biomarkers between the SARS-CoV-2 pathogenesis and recovery stage from a molecular and cellular standpoint. We found that COVID-19 patients had a much larger number of DEGs than recovered humans and healthy controls and that some of these were co-expressed in both COVID-19 patients and recovered humans. We used gene expression analysis with the biomarker to identify cellular signaling pathways and GO terms. In the COVID-19 patients, we found several genes coding for translational activities, transcription factors, hub-proteins, and miRNA expressions, all of which indicated a persistent inflammation and cytokine storm. The signaling pathways, GO terms, and chemical compounds discovered in this study could help researchers figure out how genes are linked together to find possible therapeutic approaches. However, the DEGs’ direct molecular biological functions and significant pathways discovered in this study should be investigated further to understand better the mechanisms underlying the host response to SARS-CoV-2 and identify potential therapeutic targets and drug candidates for COVID-19.

## Data availability statement

The data presented in the study are deposited in the National Center for Biotechnology Information (NCBI) repository under BioProject accession number PRJNA720904.

## Ethics statement

The protocol for sample collection from COVID-19, recovered and healthy humans, sample processing, transport, and RNA extraction was approved by the National Institute of Laboratory Medicine and Referral Center of Bangladesh. Written informed consent was obtain from the participants.

## Author contributions

MNH conceived and designed the experiments, analysed data, and wrote the manuscript. MAK, MAH, MIH and MHR contributed to data analysis and interpreting results. MMHS, MAH, SA, TAB, BG, IJ, TN, and MMAM collected samples and performed sequencing. MES edited the manuscript. YA reviewed and edited the manuscript. MSK coordinated the study and performed sequencing. CZ critically evaluated the results and edited the manuscript. TI conceived and designed the experiments, coordinated the study, and critically edited the manuscript. All authors contributed to the article and approved the submitted version.

## Acknowledgments

The authors would like to thank the individuals who helped in sample collection.

## Conflict of interest

The authors declare that the research was conducted in the absence of any commercial or financial relationships that could be construed as a potential conflict of interest.

The reviewer AZS declared a past collaboration with the author MNH to the handling editor at the time of review.

## Publisher’s note

All claims expressed in this article are solely those of the authors and do not necessarily represent those of their affiliated organizations, or those of the publisher, the editors and the reviewers. Any product that may be evaluated in this article, or claim that may be made by its manufacturer, is not guaranteed or endorsed by the publisher.
